# Supplementary material for: Dietary Supplement Use among Older Cancer Survivors: Socio-Demographic Associations, Supplement Types, Reasons for Use, and Cost
Source: Nutrients. 2022 Aug 18;14(16):3402. doi: 10.3390/nu14163402 (PMC9414522; doi:10.3390/nu14163402)
Supplement: Supplementary file 1 [file nutrients-14-03402-s001.zip › nutrients-1839819-supplementary.pdf]

Table S1: Specific supplements used among older cancer survivors and their categorization.

Main Category	Included Supplements
Vitamins	Alpha Lipoic Acid; B Complex; B-Vitamins; Benfotiamine; Biotin; Vitamin C; Coenzyme Q10; Vitamin D; Vitamin E; Folic Acid; K2; Niacin; Niacinamide; Pantothenic Acid.
Multi-vitamin/mineral Preparations	AREDs/PreserVision; Calcium + Vit. D; Caltrate; Citrical + D; Ester C; Hair Skin and Nails; Men's Multi; Multivitamin; Ocuvite; Prenatal Vitamins; Selenium + Vit. E; Men's 50+ Multi; Adult 50+ Multi; Women's 50+ Multi; Women's Multi; Viactiv; Calcium, Magnesium, and Zinc Caplet.
Minerals	Calcium; Chromium; Iodine; Iron; Magnesium; Potassium; Zinc.
Herbals	Ashwagandha; Astaxanthin; Berberine; Black Cohosh; Black Currant Seed Oil; Black Seed Oil; Bosmeric-SR Complex; Boswellia; Cell Shield RTQ; Tart Cherry Supplement; Cinnamon; Cranberry; Curcumin/Turmeric; Elderberry; Garlic; Ginger, Ginkgo Biloba; Horse Chestnut Extract; Ojibwa Tea; Saw Palmetto; Pau d'Arco; Resveratrol; Nutriferon; St. John's Wort; Red Yeast Rice.
Amino Acids/Proteins	Acetyl L-Carnitine; L-Arginine; L-Lysine; Whey Protein.
Joint Preparations	Chondroitin; Collagen Peptides; Glucosamine + Chondroitin; Glucosamine; MSM; Ortho-biotic; Osteo Biflex; OsteoPrime; Strontium Boost.
Fatty Acids/Oils	Fish Oil; Flaxseed Oil; Grape Seed Extract; Krill Oil; Lecithin; Omega 3.
Pre/Probiotics	Acidophilus; Align; Beta 1,3 D Glucan; Digestive Enzymes; Fiber Gummy; Metamucil; Microbiome; Phillip's Colon Health; Plexus Bio-cleanse/pro-bio; Probiotics; Pre- & Pro-biotic combo; 7-day Cleanse; Vitafusion Fiber Well Fit.
Other	DHEA; DIM 13C; DMAE; Focus Factor; New Health Heart Savior; Melatonin; Perfect Food; Policosanol; Drenamin; Tangy Tangerine.
